# Does elevation influence the distributional patterns of tropical myxomycetes? A case study in Costa Rica

**DOI:** 10.1080/21501203.2016.1168885

**Published:** 2016-04-07

**Authors:** Carlos Rojas, Randall Valverde, Erick Calvo

**Affiliations:** a Forest Resources Unit, Engineering Research Institute, University of Costa Rica, San Pedro de Montes de Oca, Costa Rica; b Department of Agricultural Engineering, University of Costa Rica, San Pedro de Montes de Oca, Costa Rica

**Keywords:** Biogeography, Central America, distribution, gradients, myxogastrids, Neotropics

## Abstract

In order to test the hypothesis that elevation may be an important factor accounting for the distribution of myxomycetes in tropical forests, this project was designed and conducted in Costa Rica. Two lower elevational belts were selected for this work due to their floristic and structural resemblance. Using the moist chamber technique, 40 different sites located in four different transects in two different macroclimatic regions were surveyed using three substrates during the rainy and the dry periods of 2014 and 2015. The results showed a lack of differences in diversity-based estimators according to elevation using different approaches and taxonomic differences were found across transects, collecting periods and substrates but not in relation with elevation either. Our results suggest that when increased spatial sampling resolution is implemented and floristic elements are common, elevation may not be as important of a factor in shaping the distribution of myxomycetes in tropical forests as commonly believed.

## Introduction

Myxomycetes (also known as myxogastrids or slime moulds) comprise a group of amoeboid protists (see Pawloski and Burki 2009) known to occur all over the world (Rollins and Stephenson 2011). Even though their study has primarily been taxonomic in nature, in recent years, there has been an accumulation of ecological information about the group.

In tropical studies carried out recently, elevation has been mentioned as one of the factors shaping the gradients of diversity in myxomycetes. For instance, most of the Neotropical studies have found a pattern of decreasing diversity with increasing elevation (Stephenson et al. 1999, 2004; Schnittler and Stephenson 2000; Rojas and Stephenson 2008). Even comprehensive analyses such as the work of Rojas et al. (2010) have found elevation to explain most of the variations associated with the distribution of abundant species in Costa Rica. However, in most of these cases, elevational ranges have been broad, across vegetational units and not necessarily comparable. Since ecosystem characteristics are naturally different between lower and upper elevations, and vegetational changes occur naturally along a gradient, observed elevational patterns may simply be a by-product of structural and floristic differences of the habitats and thus may not occur within a homogeneous elevational unit.

In this manner, in order to explore in more detail the evidence showing ecological and taxonomic differences at different elevations, more comprehensive and better designed studies on myxomycetes are required. Such approach would minimize potential errors coming from poor elevational resolution, unequal sampling, “snapshot” surveys and geographical site effects (see other problems in Rollins and Stephenson 2011). When those aspects are considered, the question on whether myxomycetes truly show different assemblage structures at different elevations is clearly still partially unanswered. For this reason, the present study has been designed with the objective of studying the characteristics of myxomycete assemblages associated with different elevations within homogeneous floristic units and different climates in the first elevational kilometre of Costa Rica.

Even though studies in the low elevational range have been carried out recently in the Southeast Asian tropics (e.g. Dagamac et al. 2014), the present work represents an unique point of comparison with spatially and temporally collected data from a Neotropical context. Since a good volume of information already exists for Costa Rica, this work also has the potential of putting in perspective all accumulated knowledge of myxomycetes for such territory under an elevational perspective.

## Materials and methods

This study was conducted in Costa Rica between 2014 and 2015 at the Forest Resources Unit (ReForesta) of the University of Costa Rica. The morphological concept of species was used and the taxonomic treatment followed Lado (2005–2015).

### Characteristics and location of study areas

Costa Rica is divided longitudinally from northwest to southeast by a series of mountain ranges that create two geographical slopes known as the Pacific and the Caribbean. The first one is located on the western side of the country facing the Pacific Ocean, and the second one is located on the eastern side facing the Caribbean Sea. This geographical division strongly influences the climatic patterns forcing a large number of organisms to show very distinct taxonomic structures between the two slopes. Since the central lands are located in higher parts of the country, elevational gradients from the inner section to either coast occur in most of the territory.

In addition to the latter, a very distinct yearly climatic pattern is observed in Costa Rica. This is not only influenced by the tropical position of the country but by other elements such as the annual latitudinal movement of the Intertropical Convergence Zone, the strength oscillations of the east trade winds and the orbital position of the earth in different times of the year. During the boreal winter and spring, most of the Costa Rican territory is warmer and dryer and during the boreal summer and fall, the country is cooler and moister.

When the latter two geoclimatic characteristics along with biogeographical patterns are combined, the result is a series of microclimates that provide specific conditions for the occurrence of different species assemblages within each taxonomic group present in the country. As such, not only biological diversity is high, but also system complexity due to the contextual elements of each microclimatic set-up. For this reason, in the present study, only the basal and the lower sections of the premontane elevational belts were considered during the design given the fact that their bioclimatic and floristic conditions are more similar between each other than when either one is compared with higher elevation belts such as montane forests.

**Figure 1.**
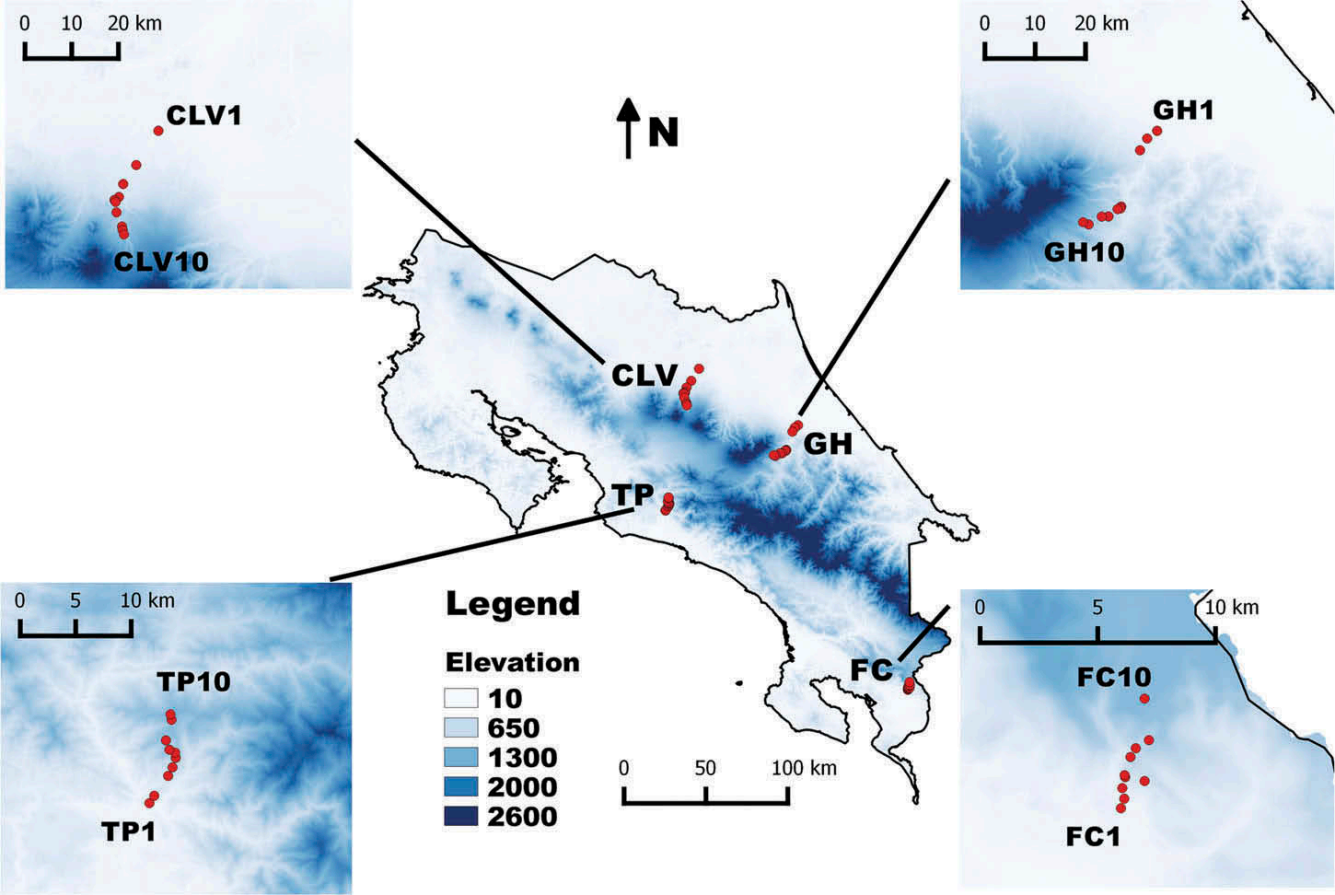
Map of Costa Rica showing the general location of all four study areas including details of the four transects established for this project. For abbreviations, see Materials and Methods section.

Four study areas were selected in the country, out of which two were located on the Caribbean and two on the Pacific slope in order to have two replicates per biophysical region. The first ones were geographically situated on the north-eastern sides of Turrialba and Barba Volcanoes, whereas the second ones were located on the southern side of the Fila de Bustamante and Fila de Cal mountains. In each area, an elevational transect between 100 and 1100 m was designed and 10 sampling points per transect were set up every 110 m in elevation using a digital altimeter (Figure 1). In this manner, the two Caribbean transects corresponded to the Guayabo de Turrialba-La Herediana de Siquirres section (hereafter referred to as GH) located between 10.13490 N/83.55450 W and 9.96933 N/83.68881 W and the Cinchona de Sarapiquí-La Virgen de Sarapiquí section (referred to as CLV) located between 10.44769 N/84.10264 W and 10.24503 N/84.17008 W. Similarly, the two Pacific slope transects corresponded to the Teruel de Acosta-Punto Caspirola de Acosta section (referred to as TP) located between 9.66208 N/84.28864 W and 9.73506 N/84.27133 W and the Fila de Cal section (referred to as FC) between Ciudad Neily and San Vito located between 8.67015 N/82.94589 W and 8.86065 N/82.93678 W.

### Sampling and recording

In all 10 sites of each transect, a series of 15 substrate samples were collected during both the rainy season in September 2014 and the dry season in February 2015. Substrate samples were divided into five ground litter (GL), five aerial litter and five twig samples for a total of 50 samples of each substrate per transect per collecting period. In this way, each transect was surveyed on the basis of 150 samples per collecting period for a grand total of 1200 samples for the complete study.

Each sample was used to set up a moist chamber culture using the protocol provided by Stephenson and Stempen (1994). With this method, the experimental material is placed on top of a piece of filter paper previously placed in a Petri dish. Water is then added until the material is covered and left in the dish for 24 h, after which the excess is poured off the dish. The material is then carefully examined for positive signs of myxomycete activity for a period of 2–3 months in which additional water is added when the culture shows signs of dryness in order to keep a high moisture microenvironment inside at all times. Since pH has been observed to be one determinant factor driving the occurrence of myxomycete species, this variable is determined within the first 2 days after the experimental material is soaked in the initial water treatment.

For this study, when positive signs of myxomycete activity were noted, in the form of plasmodia, plasmodial tracks or fruiting bodies, the observation was recorded. In the last case, however, fruiting bodies were extracted from the culture using fine forceps and glued into a match-sized pasteboard box. All collections were identified upon extraction, placed in a 3-week quarantine period in a cold chamber at 2°C and deposited in the Myxogastrid Biorepository of the Engineering Research Institute at the University of Costa Rica for future reference.

### Analyses

General descriptive statistics were used to summarize the data obtained in this project, and hypothesis-based approaches such as contingency table analyses, comparisons of means and analyses of variance were used when necessary. In the latter cases, normality of the quantitative variables was tested before the technique was used and the cut-off probability value for rejecting the null hypothesis was 0.05. A generalized mixed linear model was also performed on myxomycete abundance considering region, transect, collecting period and substrate as random effects in order to evaluate the effect of elevation on a diversity estimate. In these cases, the software JMP, v. 10 was used.

In addition, the abundance of records for all species was organized in a series of five overall abundance categories established with the structure of the data set. For this part, those species with relative abundance values ˃10% were classified as very abundant (VA), between 3% and 10% as abundant (A), between 0.3% and 3% as common (C), between 0.1% and 0.3% as occasional (O) and ˂0.1% as rare (R). Testing of the relative abundance structure of the myxomycete assemblages according to different variables was performed using a null model of equal probability for all levels within the variables studied. For the latter, only species in the VA and A categories were considered.

For the elevation analyses, a reclassification of the actual elevations for all sites in all transects was performed before testing differences among levels. Using this approach, elevations were rearranged in three categories: “high”, “medium” and “low” for the 1100–800, 799–450 and 449–100 elevational sections, respectively. In a similar way to the other cases, differences across the elevational gradient were tested under the null model of equal probability at all levels of analysis for each variable under study.

For biodiversity purposes, an index-based approach was taken. In this case, both the Shannon–Wiener and the intuitive 1-D Simpson indices of diversity were calculated, and t-test analyses were used for different comparisons. In addition, the taxonomic diversity index (TDI) (number of species/number of genera) was calculated for the overall data set. Also, beta diversity analyses were performed for the comparisons among transects and across elevation categories with hierarchical cluster analyses using Bray–Curtis distances as suggested by Rojas et al. (2012). In all these cases, the software PAST, v. 3.09 (Hammer et al. 2001) was used.

## Results

A total of 2043 records of myxomycetes were obtained with the 1200 moist chambers analysed during this project. Cultures observed to be positive for myxomycete activity represented 69% of the total number of moist chambers, whereas 65% of the same group showed actual fruiting bodies. A total of 67 species in 16 genera were identified from all collections (see Table 1) with an overall TDI value of 4.2. The most commonly recorded species were *Arcyria cinerea, Didymium squamulosum* and *Lamproderma scintillans*.

**Table 1.** Myxomycete species and number of records according to overall abundance categories (OAC) and transects studied in the present investigation.

			Transect			
			Atlantic region		Pacific region	
Species/diversity estimator	pH	OAC	CLV	GH	FC	PT
*Arcyria cinerea*	6.7	VA	95	57	90	55
*Didymium squamulosum*	6.8	VA	37	33	113	96
*Lamproderma scintillans*	6.8	VA	45	57	64	92
*Diderma hemisphaericum*	6.8	A	46	68	30	47
*Didymium difforme*	6.5	A	32	39	31	36
*Physarum pusillum*	6.7	A	27	21	21	22
*Comatricha tenerrima*	6.6	A	14	25	16	26
*Physarum compressum*	7.1	A	4	10	43	17
*Perichaena chrysosperma*	6.9	A	11	25	14	18
*Didymium bahiense*	6.5	C	10	5	21	15
*Stemonitis fusca*	6.2	C	11	4	12	19
*Arcyria afroalpina*	6.9	C	2	6	16	14
*Didymium minus*	6.7	C	8	8	6	10
*Didymium iridis*	6.6	C	9	4	13	4
*Comatricha nigra*	6.7	C	5	9	7	7
*Didymium clavus*	6.7	C	12	6	0	8
*Diderma effusum*	7.0	C	1	0	25	0
*Perichaena depressa*	7.0	C	6	10	5	3
*Diachea leucopodia*	5.9	C	5	0	4	13
*Perichaena pedata*	7.2	C	2	9	8	2
*Physarum decipiens*	6.7	C	6	3	6	1
*Physarum didermoides*	7.4	C	1	8	5	1
*Physarum oblatum*	6.5	C	7	1	5	0
*Physarum nucleatum*	6.3	C	8	1	0	1
*Willkommlangea reticulata*	6.0	C	2	0	1	6
*Didymium dubium*	7.0	C	0	6	1	1
*Physarum cinereum*	6.8	C	1	1	4	2
*Physarum melleum*	6.3	C	0	2	1	5
*Physarum album*	6.5	C	0	0	4	3
*Comatricha subcaespitosa*	5.9	O	0	1	5	0
*Didymium nigripes*	7.0	O	1	2	3	0
*Perichaena vermicularis*	6.6	O	1	0	3	2
*Physarum bivalve*	6.4	O	0	1	5	0
*Comatricha pulchella*	5.7	O	0	2	1	2
*Arcyria denudata*	6.8	O	2	0	2	0
*Comatricha laxa*	6.8	O	0	0	5	0
*Paradiacheopsis longipes*	5.6	O	0	0	1	3
*Physarum leucopus*	6.6	O	4	0	0	0
*Arcyria globosa*	6.5	O	0	2	0	1
*Collaria arcyrionema*	7.5	O	0	0	3	0
*Comatricha elegans*	5.9	O	0	0	1	2
*Cribraria violacea*	7.2	O	1	0	2	0
*Didymium anellus*	6.4	O	1	2	0	0
*Physarum auriscalpium*	6.9	O	0	1	2	0
*Physarum bogoriense*	6.4	O	1	0	1	1
*Physarum roseum*	6.9	O	2	0	0	1
*Physarum viride*	6.9	O	3	0	0	0
*Hemitrichia pardina*	6.9	R	5	9	3	10
*Physarum aeneum*	6.9	R	0	0	2	0
*Physarum mutabile*	6.9	R	0	0	2	0
*Physarum stellatum*	6.7	R	1	0	1	0
*Physarum superbum*	7.0	R	0	1	0	1
*Stemonitopsis amoena*	5.8	R	0	0	0	2
*Arcyria pomiformis*	5.5	R	0	0	1	0
*Comatricha lurida*	6.9	R	0	0	1	0
*Comatricha suksdorfii*	7.4	R	1	0	0	0
*Cribraria tenella*	5.7	R	0	0	0	1
*Didymium melanospermum*	7.2	R	0	0	1	0
*Didymium serpula*	7.4	R	0	0	1	0
*Diachea bulbillosa*	7.7	R	0	0	1	0
*Hemitrichia calyculata*	6.9	R	0	0	0	1
*Hemitrichia serpula*	7.3	R	0	0	0	1
*Licea pusilla*	7.3	R	0	0	1	0
*Perichaena corticalis*	8.3	R	0	1	0	0
*Physarum galbeum*	4.7	R	0	0	1	0
*Stemonitis uvifera*	4.5	R	0	0	0	1
*Stemonitopsis hyperopta*	7.2	R	0	1	0	0

Average pH values are provided in all cases. For abbreviation, see Materials and Methods section.

For all studied samples, pH was normally distributed between 3.5 and 8.6 (mean = 6.7, standard deviation = 0.6), but significant differences were found by region (*t *= −3.0, *df *= 2041, *P *= 0.001), season (*t *= −10.2, *df *= 2041, *P *= 0.0001) and substrate types (*F*(2,2040) = 80.2, *P *= 0.001) with lower pH values on the Pacific slope, during the dry season and on twigs (TW), respectively. Interestingly, aerial litter yielded significantly more myxomycetes than either GL or TW (*χ*^2^ = 203.2, *df* = 2, *P *= 0.0001 for the complete comparison and *χ*^2^ = 1.3, *df* = 1, *P *= 0.25 for the GL-TW comparison). Also, positive results showed to be different from a null model of equal probability of incidence for all levels of study for tests on seasons (*χ*^2^ = 26.1, *df* = 1, *P *= 0.0001), regions (*χ*^2^ = 43.1, *df *= 1, *P *= 0.0001) and transects (*χ*^2^ = 47.3, *df *= 1, *P *= 0.0001), with myxomycetes being more commonly recorded during the dry season, on the Pacific slope and in either the FC or TP transects (no difference between these two, not shown). The Whittaker index beta diversity value for the complete treatment arrangement was 0.61.

**Figure 2.**
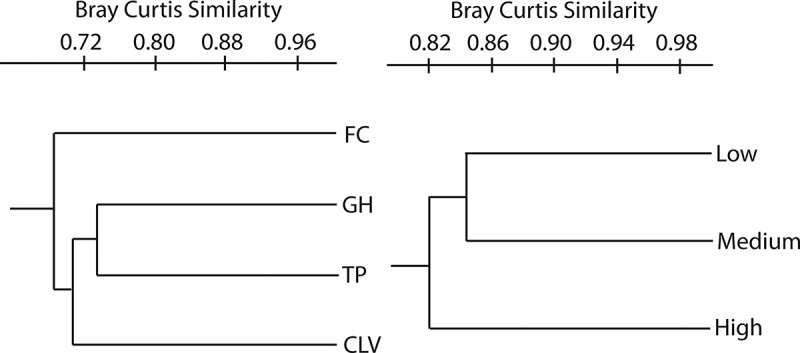
Bray–Curtis-based hierarchical cluster trees showing the taxonomic and structural proximity of transects (left) and elevation categories (right) used during this project. All branching are supported by bootstrap values higher than 0.50.

The taxonomic structure of each transect was naturally different for the lower abundance categories. However, differences in the abundance of the “very abundant” species were observed across transects (all *P* values under 0.0001), but no differences were observed in the “abundant” category, except for *Physarum compressum*. In all cases, the highest diversity values were observed in the Pacific slope transects with the FC transect providing most of the differences (see Table 2). The GH and TP transects were the most similar to each other, with CLV closer to either one. The most taxonomically dissimilar transect was FC according to the Bray–Curtis distance analysis (Figure 2).

**Table 2.** Diversity-based estimator values according to transects and elevational categories used in the present investigation.

Categories	ONS	Simpson’s DI	Shannon’s DI	Evenness	MNS (Chao 1)
Transects					
CLV	38	0.90	2.83	0.44	45
GH	36	0.91	2.84	0.47	42
FC	51	0.91	2.99	0.39	68
TP	41	0.91	2.83	0.41	51
Elevation					
Low	56	0.92	3.07	0.38	78
Medium	47	0.91	2.87	0.37	65
High	47	0.92	2.85	0.40	55

ONS: observed number of species; MNS: maximum number of species.

**Figure 3.**
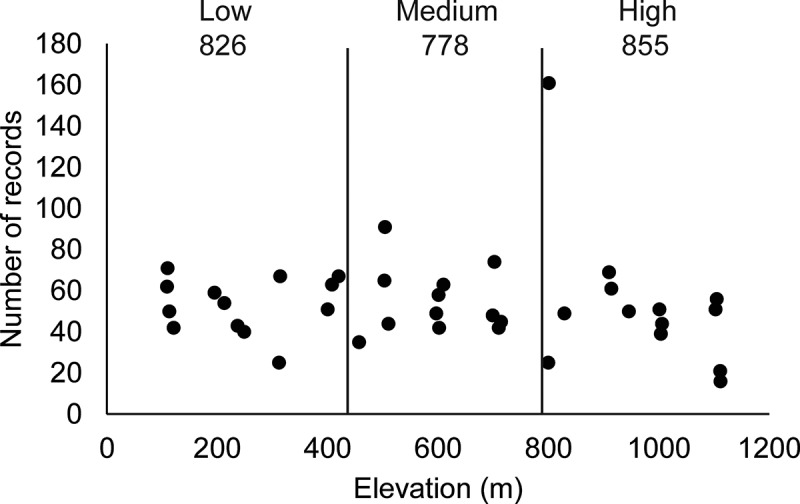
Scatter plot of abundance values for all 40 sampling sites studied during the present investigation arranged according to elevation. Elevational categories and abundance values are also shown. A lack of trend and correlation between the two variables is noted, even though an outlier around 800 m is present.

No differences across elevational categories were found when total abundance was considered (Figure 3, *χ*^2^ = 3.69, *df* = 2, *P *= 0.15), and no strong correlation between elevation and abundance was observed (*r*^2^ = 0.44). In the mixed model performed, the restricted maximum likelihood matrix did not show any level of the analysis to account for more than 8% of the total variation, and the fixed effect (elevation) did not show any significant differences (*P *˃ 0.05) at any level. At the taxonomic level, for the “very abundant” and “abundant” species, no differences in the elevation categories were observed, except for *D. squamulosum*, which showed a higher frequency at medium elevations. The taxonomic resemblance among myxomycete assemblages was found to be higher for the medium–low elevation category than for either the medium–high or low–high comparisons (Figure 2). However, no differences in the diversity indices were found among the three elevational categories (see Table 2, *t *= −1.78, *df *= 1319, *P *= 0.08 for the low–medium comparison, *t *= 1.29, *df *= 1316, *P *= 0.19 for the medium–high comparison, *t *= −0.85, *df *= 1386, *P *= 0.39 for the low–high comparison). The Whittaker index values of beta diversity for elevational categories showed values between 0.20 and 0.25 for all combinations.

## Discussion

The productivity of moist chambers from the present study is comparable to previous research from Costa Rica (e.g. Schnittler and Stephenson 2000). Also, the diversity values calculated in the present study are similar to published research in the Neotropics with a similar effort (e.g. Rojas and Stephenson 2012). Interestingly, for Southeast Asia, productivity associated with myxomycete surveys along elevational transects of similar conditions has been reported to be lower (about 23%, see Dagamac et al. 2012). Whether this is a firm pattern or simply the effect of multiple conditions affecting the development of research is still a question that warrants study. Unpublished results obtained by the first author in northern Thailand showed intermediate values (about 50%) and as observed in the present study and others, there may be differences in the yields obtained from moist chamber isolation according to a number of variables in the study. For example, collecting period and structural differences of the study areas (see Rojas and Stephenson 2012) generate differences in moist chamber culturing.

Interestingly, dryer periods and aerial litter are one more time associated with higher abundance and diversity values. This is a common pattern in myxomycete surveys worldwide. As discussed by various authors in recent decades (see Stephenson et al. 2004), even though dead leaves on the ground and those still attached to the plant are equivalent in several ways, it seems that exposure to wind associated with less homogeneous microclimatic conditions makes aerial substrates more productive during myxomycete laboratory isolation protocols. In the case of the present study, it is interesting to note, however, that all transects showed similar abundance and diversity values within their respective climatic region, but the myxomycete assemblage associated with the FC area was the one that showed the most differences in all comparisons. In recent years, this area of Costa Rica has been studied in more detail, and a number of new records for the country have been found in their forests (see Rojas et al. 2015), presumably as a consequence of historical, geological and biogeographical reasons (see Clement and Horn 2001 for a discussion on land use in that part of Costa Rica during the last 3000 years) that have increased the mosaic of microenvironments available.

The differences recorded in the present study both at the diversity and at the taxonomic level seem to be associated with macroclimatic and microclimatic conditions, quality of substrate and structural conditions of the forest. This is similar to what has been reported in a similar study in the Philippines (Dagamac et al. 2012). However, no differences according to elevation were found during this investigation, and the effect of region, transect, substrate and collecting period along with their intersects was found to be very minor in a mixed model evaluating record abundance. As mentioned before, a number of studies in Neotropical areas (Stephenson et al. 1999, 2004; Schnittler and Stephenson 2000; Rojas and Stephenson 2008) had reported a pattern of decreasing diversity with increasing elevation. However, those studies were based on broader elevation ranges across different elevational belts and vegetation units. As such, it seems that the pattern still holds for those conditions but maybe not within homogeneous floristic and elevational units as observed in the results from the present study. The logical consequence of this is that in a similar manner to other organisms, myxomycetes may show preference for particular conditions but can still survive in a range of variables according to the particular species-level ecological plasticity.

One interesting result is that the only species found to be associated with an elevational variable was *D. squamulosum*. This morphospecies is known to have a wide distribution in the Neotropics (see Lado and Wrigley de Basanta 2008) and thus, the logical interpretation is that it represents an entity able to occur across a wide range of ecological conditions. However, our results suggest that some factors may be accountable for the elevational differences, particularly in the FC transect, where most of the records for the species were found. It is interesting to note that this was the only area with limestone (observed in the high levels of Calcium) where the slope reached values close to 45° in some sections and where condensation is highly favourable due to the rapid change in elevation. The latter still does not explain the unusual high occurrence of *D. squamulosum* but may suggest that microenvironmental conditions not tested in the present study could be responsible for those differences as seen in a number of previous researches (e.g. Schnittler and Stephenson 2000).

In any case, the results presented in this study suggest that higher ecological definition may be a key factor for the design of future field-based and hypothesis-driven studies on myxomycetes. As observed herein, previous published patterns may occur at certain spatial or temporal scales but not at others, and so, different research designs have the potential of bringing a different perspective on the ecology of a group of organisms for which there is still plenty of research to be conducted. The higher ecological complexity of tropical systems also makes the interpretation of results difficult, since by default all investigations are conducted under very particular circumstances. In this manner, the accumulation of studies showing defined patterns in different settings is a particularly good strategy in the tropics to generate empirical conclusions. However, those patterns cannot be found unless basic research, at different scales, is still carried out across the range of ecological conditions where myxomycetes are found.
